# The limits of convergence in the collective behavior of competing marine taxa

**DOI:** 10.1002/ece3.8747

**Published:** 2022-03-22

**Authors:** Benjamin P. Burford, R. Russell Williams, Nicholas J. Demetras, Nicholas Carey, Jeremy Goldbogen, William F. Gilly, Jeffrey Harding, Mark W. Denny

**Affiliations:** ^1^ Hopkins Marine Station of Stanford University Pacific Grove California USA; ^2^ Institute of Marine Sciences, affiliated with the National Oceanic and Atmospheric Administration National Marine Fisheries Service Southwest Fisheries Science Center University of California Santa Cruz Santa Cruz California USA; ^3^ 236780 Marine Scotland Science Aberdeen UK; ^4^ National Oceanic and Atmospheric Administration National Marine Fisheries Service Southwest Fisheries Science Center Santa Cruz California USA

**Keywords:** cephalopod, collective motion, evolutionary convergence, fish, interaction rules

## Abstract

Collective behaviors in biological systems such as coordinated movements have important ecological and evolutionary consequences. While many studies examine within‐species variation in collective behavior, explicit comparisons between functionally similar species from different taxonomic groups are rare. Therefore, a fundamental question remains: how do collective behaviors compare between taxa with morphological and physiological convergence, and how might this relate to functional ecology and niche partitioning? We examined the collective motion of two ecologically similar species from unrelated clades that have competed for pelagic predatory niches for over 500 million years—California market squid, *Doryteuthis opalescens* (Mollusca) and Pacific sardine, *Sardinops sagax* (Chordata). We (1) found similarities in how groups of individuals from each species collectively aligned, measured by angular deviation, the difference between individual orientation and average group heading. We also (2) show that conspecific attraction, which we approximated using nearest neighbor distance, was greater in sardine than squid. Finally, we (3) found that individuals of each species explicitly matched the orientation of groupmates, but that these matching responses were less rapid in squid than sardine. Based on these results, we hypothesize that information sharing is a comparably important function of social grouping for both taxa. On the other hand, some capabilities, including hydrodynamically conferred energy savings and defense against predators, could stem from taxon‐specific biology.

## Graphical abstract

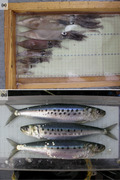

## 1 INTRODUCTION

Collective motion in groups of social animals, such as swarms of insects, flocks of birds, and schools of fish, arises from interactions between individuals. By sensing and responding to the behaviors of proximate groupmates, individuals of such species can act in a coordinated fashion with little or no sense of the group’s behavior. In doing so, they often benefit from group capabilities that are much more limited at the level of individuals. These include improved defense through reduced predation risk (Ioannou, 2017), more efficient navigation through opinion pooling (Berdahl et al., 2018), and reduced energy expenditure through hydrodynamic or aerodynamic effects (Marras et al., 2015).

The collective benefits gained often depend on how groups organize during collective motion (MacGregor et al., 2020). For example, the flow of social information between individuals that underlies responses to threats by fish schools is enhanced when they adopt highly polarized organization (Ioannou et al., 2011). While this allows for rapid group responses to environmental stimuli (Makris et al., 2006), it has the drawback of increasing the group’s susceptibility to false alarms (Ioannou et al., 2011). Conversely, poor group organization can lead to the loss of potentially valuable social information such as the location of food resources (MacGregor et al., 2020).

Group organization during collective motion can be characterized by collective alignment—that is, directional organization, or how individuals move together in the same direction, and conspecific attraction—that is, spatial organization, or how individuals move together with consistent spacing (MacGregor et al., 2020; Schaerf et al., 2017), and these characteristics are maintained via interaction rules (Herbert‐Read, 2016). However, there is considerable between‐ and within‐species variation in these metrics—a fundamental goal in collective behavior research is to understand how this variation is related to functional ecology (Sumpter et al., 2018).

Within‐ and between‐species variation in alignment and attraction, and the relevant interaction rules, can reflect differences in sensory modality and locomotion (Herbert‐Read, 2016; Schaerf et al., 2017). For example, the Mexican tetra (*Astyanax mexicanus*) tends to maintain close alignment and attraction to groupmates, but the blind cave‐dwelling form of this species does not, even though it possesses an enhanced pressure‐sensing lateral line (Kowalko et al., 2013). In one of the few studies to directly compare the collective motion of different species with standardized methodology, Partridge et al. (1980) suggested that attraction during collective motion could reflect maneuverability. The authors found that individuals in groups of cod (*Gadus morhua*) or saithe (*Pollachius virens*) swam closer together, while the less maneuverable species, herring (*Clupea harengus*), swam further apart.

In evolutionary convergence, unrelated taxa develop analogous characteristics in response to similar selective pressures. Perhaps the most well‐known examples are of convergent morphology and physiology relevant to sensing and locomotion (Donley et al., 2004; Nilsson, 2009). Increasingly, convergence in social behavior is also recognized (Barsbai et al., 2021; Weilgart et al., 1996). To our knowledge, convergence in collective motion between unrelated taxa that share a functional and ecological role within the same ecosystem has not been directly investigated.

A striking example of evolutionary convergence is that between cephalopods (Mollusca) and fish (Chordata) in the ocean. Cephalopod and fish convergence reflects over 500 million years of competition for pelagic predatory niches, which was initiated through the independent evolution of locomotion strategies (jet propulsion in cephalopods, fin undulation in fish) that enabled both groups to exploit the water column of the oceans (Klug et al., 2010; Packard, 1972). Convergent morphological and physiological adaptations are particularly evident between squid (Cephalopoda) and fish—these include fins for swimming, image‐forming camera eyes, calcium carbonate‐based equilibrium organs, pressure‐sensing lateral lines, and communicative pigmentation patterning (Budelmann & Bleckmann, 1988; Clarke, 1966; Hanlon & Messenger, 2018; Packard, 1972; Pavlov & Kasumyan, 2000). While the functions of these adaptations are similar, being independently evolved, their structures can be quite different because they stem from taxon‐specific biology (O’Dor & Webber, 1986).

About 50% of fish species are known to live in groups for at least part of their lives, and many of these species exhibit collective motion (Pavlov & Kasumyan, 2000). For squid species that have been studied in sufficient detail (e.g., commercially important, epipelagic, and/or nearshore species), 82% (58 species) are known to form social groups (Burford et al., 2019; Burford & Robison, 2020; Jereb & Roper, 2010). Of these social squids, some are capable of collective motion, as evidenced by video, laboratory, and acoustic studies of group organization and observations of coordinated group movements (Adamo & Weichelt, 1999; Benoit‐Bird & Gilly, 2012; Hurley, 1978; Mather & O’Dor, 1984; Moynihan & Rodaniche, 1982; Sugimoto & Ikeda, 2012; Sugimoto et al., 2013).

Alignment and attraction metrics, including angular deviation and nearest neighbor distance, respectively, measured for squid are generally similar to published values for fish (see previous references). This similarity suggests squid exhibit comparable collective organization. However, lack of standardization in procedures or analytical methods prevents more detailed comparison. More recently, standardized acoustic techniques have confirmed broadly similar attraction (mean interindividual distance) of mono‐specific squid and fish social aggregations in deep scattering layers (Benoit‐Bird et al., 2017). In addition, threat‐related information appears to be transferred between such aggregations, suggesting that between‐taxa information sharing could be a critical function of similarity in collective organization (Benoit‐Bird et al., 2017). Such work provides the rationale for a detailed comparison of squid and fish collective behavior between co‐occurring, functionally similar species.

**FIGURE 1:**
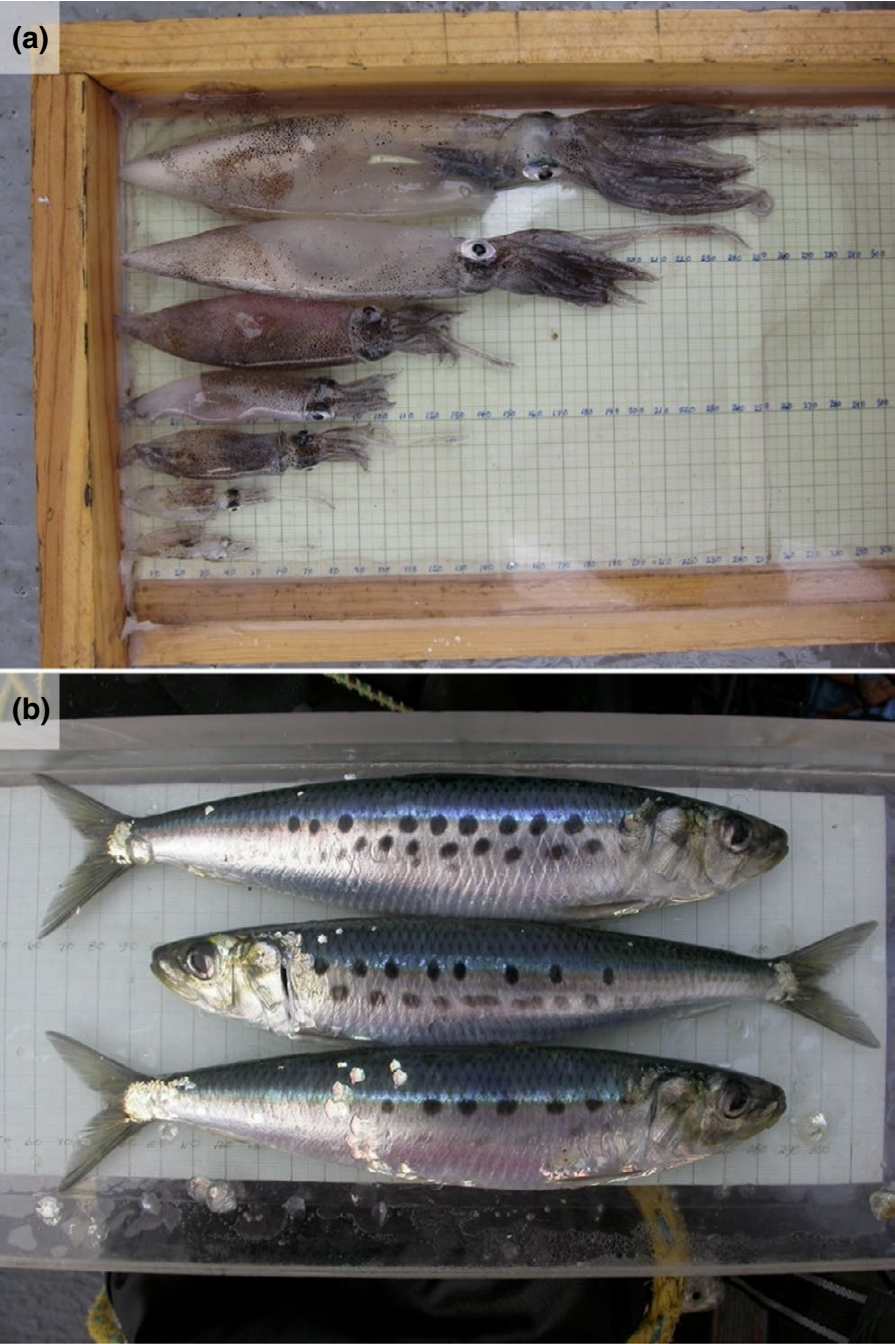
Trawl‐caught specimens of California market squid and Pacific sardine. Photo credits: Jeffrey Harding

Here, we examined the convergence in collective motion between competing squid and fish species: California market squid (*Doryteuthis opalescens*) (Figure 1a) and Pacific sardine (*Sardinops sagax*) (Figure 1b). These species inhabit the same ecosystem, the California Current System (CCS), where they overlap in time and space and are ecologically similar. Both are highly migratory (Burford et al., 2022; Checkley et al., 2009; Payne & O’Dor, 2006; Zeidberg, 2004), primarily feed on the same small pelagic crustaceans (Burford et al., 2022; Miller & Brodeur, 2007; Zeidberg, 2013), and are a significant food resource for an assortment of upper trophic level species including whales, dolphins, seabirds, pinnipeds, tunas, and sharks (Jereb & Roper, 2010; Whitehead et al., 1988). Like many pelagic fishes and squids, each species lives in social aggregations throughout much of their lives, and this behavior likely serves critical functions in their migration, feeding, and predation risk (Ritz et al., 2011).

**FIGURE 2:**
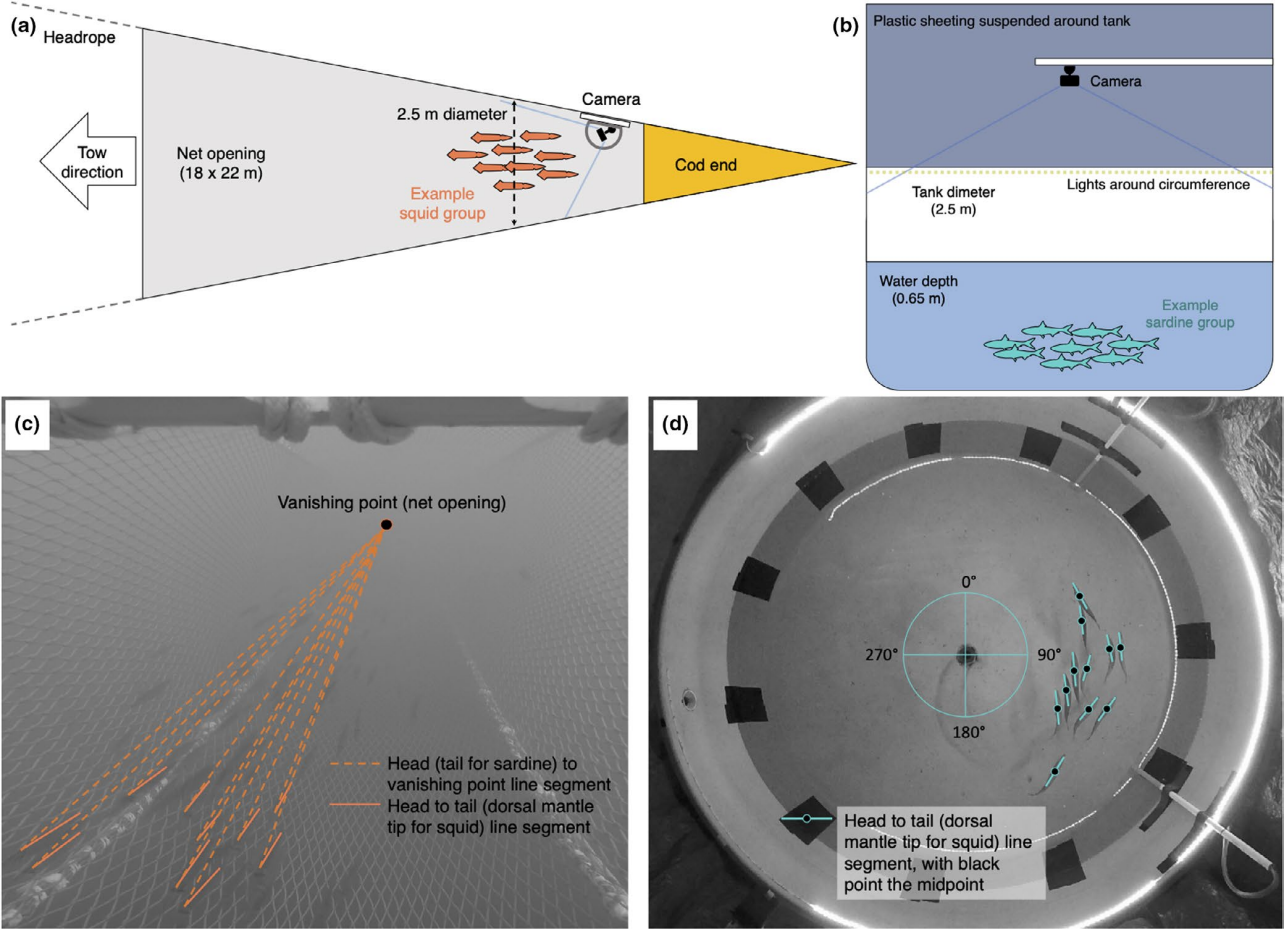
Measuring the collective organization of moving sardine or squid groups in situ and in the laboratory. (a) Diagram of net trawl setup for recording footage of swimming groups of both species in situ (not to scale). (b) Diagram of tank setup for recording footage of swimming groups of both species in the laboratory (not to scale). (c) Illustrative example of an angular deviation (collective alignment metric) frame analysis for a squid group measured in situ. The line segments connecting individuals to the vanishing point are dashed and the line segments along the lengths of individuals are solid. The angle formed by each solid‐dashed line pair is an individual’s orientation; angular deviation (°) is the difference between each individual’s orientation and the average group orientation. Animal position within the camera’s field of view may have changed the possible length of the line segment drawn along its body, but it did not obscure the vanishing point, and thus reliable orientations could be consistently determined. (d) Illustrative example of a nearest neighbor distance (NND, conspecific attraction metric) frame analysis for a sardine group measured in the laboratory. The midpoint of the solid line running along the length of an individual is the centroid; NND is the shortest distance to another groupmate’s centroid. NND is calculated in terms of a group’s average body length. Angular deviation frame analysis for laboratory footage used an individual’s orientation with respect to default image degree coordinates, as illustrated in (d)

To compare collective motion between California market squid and Pacific sardine, we used standardized methodologies both in the field and in laboratory. In the field, observations were recorded in a large trawl net that was towed at speeds comparable to fast swimming in each species (Figure 2a), while laboratory observations were collected in a large, shallow, circular tank with minimal flow (Figure 2b). Although much slower swimming speeds were exhibited in the latter treatment, both scenarios likely led to increased vigilance, as they are non‐natural situations that would alarm individuals. Previous research has shown that group organization tends to increase under alarming situations (Schaerf et al., 2017), and that collective decision‐making can differ between populations from high‐ versus low‐risk environments (Herbert‐Read et al., 2019). Thus, our investigation examines how groups of each species organize during collective motion in somewhat confined environments under heightened vigilance. To do so, we compared alignment, attraction, and an alignment‐based interaction rule regarding how individuals respond to spontaneous changes in the orientation of groupmates that could serve to coordinate collective movements. Based upon the species’ ecologies and previous research, we hypothesized that: (1) both species would exhibit close alignment and attraction during collective behaviors; (2) each species would similarly conform to the alignment‐based interaction rule.

## 2 MATERIALS AND METHODS

### 2.1 Data collection

#### 2.1.1 In situ observations

In situ footage of moving groups of sardine and squid were collected in the CCS using a GoPro Hero 3 action camera (GoPro Inc.) mounted in a 264 Nordic Rope Trawl (Figure 2a, Videos S1 and S2) operated off the *R*/*V Ocean Starr* during NOAA National Marine Fisheries Service scientific surveys of juvenile salmon abundance and distribution in September 2015. Trawls were conducted at sampling stations throughout the CCS. Based on video quality, we selected footage of 5 groups of each species captured during hauls off northern California (Table 1). Trawls were conducted according to methods described previously (Harding et al., 2011, 2021). This work was conducted under NOAA project OS1503.

**TABLE 1:** In situ footage and corresponding sardine and squid group metadata

Species	Group	Duration (s)	Date	Latitude (°N)	Longitude (°W)	Bottom depth (m)	Distance offshore (nm)	Group size (count)	Average length (mm)	SD length (mm)	Length subsample (*n*)
Squid	1	17	9/8/15	41.5833	−124.2533	51	7	55	75.16	10.97	32
Squid	2	18	9/12/15	39.25	−123.8292	92	2	88	72.52	6.58	29
Squid	3	32	9/12/15	39.25	−123.8292	92	2	130	72.52	6.58	29
Squid	4	32	9/12/15	39.25	−123.8292	92	2	162	72.52	6.58	29
Squid	5	17	9/10/15	40.6333	−124.5133	700	8.8	141	80.33	8.76	30
Sardine	1	16	9/13/15	38.5	−123.26	55	1.8	192	119.35	7.08	20
Sardine	2	14	9/13/15	38.5	−123.26	55	1.8	125	119.35	7.08	20
Sardine	3	201	9/13/15	38.5	−123.26	55	1.8	845	115.77	8.48	31
Sardine	4	540	9/14/15	37.8417	−122.695	28	6.6	658	116.09	5.39	32
Sardine	5	3	9/14/15	37.8417	−122.695	28	6.6	30	116.09	5.39	32

Note

Group numbering is consistent with Figure 4 and Figure S1.

The footage we analyzed was captured during daylight hours at 18–24 m depth while the net was being towed at 1.5 m s^−1^. At this speed, squid and sardine groups could temporarily keep pace with the net and swim in front of the camera, but eventually left the field of view and were collected in the end of the net (cod end, Figure 2a). When towed, the net’s opening was approximately 18 × 22 m, but the size was tapered down to approximately a 2.5 m diameter in the intermediate net section where the camera was mounted (Figure 2a). The camera faced toward the opening of the net and captured footage at 30 frames s^−1^. Although the net enclosed the groups and encouraged swimming toward the opening, it did not overly constrain motion, as dynamic coordinated moments within the net were visible in the footage (e.g., Video S2). While we cannot rule out the possibility, it is unlikely that the observed group behavior was a shared response to environmental stimuli without a collective component.

Selected footage of squid and sardine groups ranged from 17 to 32 s and from 3 to 540 s in duration, respectively. When corrected for group size, this equated to 0.12–0.31 s per individual in squid and 0.08–0.82 s per individual in sardine. We counted the number of animals in observed groups from the footage. This scoring was not possible for two large sardine groups. In these groups, we estimated the sizes from the total number of specimens collected in the respective trawls. The average size in terms of length (dorsal mantle length for squid and fork length for fish, both in mm) was determined for squid and sardine from a haphazard (i.e., no randomization method, but no selection criterion) subsample of specimens collected in each trawl. Both squid and fish tend to group with similar‐sized individuals (Benoit‐Bird & Gilly, 2012; Pavlov & Kasumyan, 2000). Because there could be multiple squid or sardine groups in a single trawl, size could only be determined for each group as the species average of subsampled specimens from each trawl (Table 1).

#### 2.1.2 In laboratory observations

We recorded footage of moving groups of sardine and squid in the DeNault wet laboratory facility at Hopkins Marine Station of Stanford University in Pacific Grove, CA (Videos S3 and S4). Squid specimens were collected near spawning grounds in the nearby waters of southern Monterey Bay, CA, from January to July 2018 using barbless jigs. Only undamaged specimens captured by the sucker cups were used in this research. Squid were held in groups in a 3200‐L circular holding tank with flow‐through seawater (20 L min^−1^) for at least 24 h before experiments. There, we fed squid small, live feeder fish (Rosy red minnow, *Pimephales promelas*) twice daily. Squid husbandry was conducted under permit Stanford IACUC #10643. Sardine were collected from a commercial bait supplier in Oxnard, CA and held under the same conditions as squid. Sardine were fed commercial fish feed four times daily (2 mm sinking pellets, Skretting, USA). This work was conducted under permit Stanford IACUC #28859 for working on fish.

Groups of each species (7–11 individuals each, Table 2) were recorded at 30 frames s^−1^ using a GoPro Hero 5 action camera (GoPro Inc.) suspended over a large circular tank (2.5 m diameter) shielded from visual disturbances by opaque plastic sheeting and lit around its circumference using LED strip lighting (Figure 2b). All effort was made to remove any visual, audio, or vibrational stimuli. The camera was remotely triggered to further avoid disturbance. All experiments were conducted at the same time of day under the same lighting conditions. Water depth in the experimental tank was 0.65 m. We supplied flow‐through seawater (10 L min^−1^) to maintain temperature (15°C) and oxygen saturation (8 mg O_2_ L^−1^) during experiments. Water was circulated clockwise at 5 cm s^−1^ to encourage sardine groups to swim in a consistent counter‐clockwise direction; the same flow treatment was also used for squid. This slight flow did not affect visibility, nor did it induce strenuous swimming behavior in either species. Squid groups used a mixture of fin undulation and jet propulsion to move back and forth within the tank, and this swimming behavior is not affected by such low flow rates (Burford et al., 2019). Between‐species differences in rheotaxis could have influenced collective organization, but this is difficult to determine given our current understanding of this process in either species.

**TABLE 2:** Laboratory footage and corresponding sardine and squid group metadata

Species	Group	Date	Duration (min)	Group size (count)	Average length (mm)	SD length (mm)
Squid	1	2/7/18	30	7	117.14	13.33
Squid	2	7/6/18	30	11	136.55	6.67
Squid	3	7/6/18	30	9	135.00	11.64
Sardine	1	3/4/18	30	11	193.27	12.54
Sardine	2	9/19/18	30	11	194.91	6.98
Sardine	3	9/19/18	30	9	192.22	11.41

Note

Group numbering is consistent with Figures 4 and 5, Figure S1.

Following an introduction to the experimental tank, groups were allowed to recover for one hour from any stress due to handling. Their behavior was then recorded for 30 min. Because this footage was collected to assess startle response latencies in related research, groups were exposed to camera‐strobe flashes once every 5 min. Strobe flashes elicit C‐starts in sardine (Video S3) and escape jets in squid (Video S4, Otis & Gilly, 1990), which we observed temporarily disrupted grouping behavior. We therefore analyzed only footage collected immediately before each strobe flash as described in the next section. Observed collective motion was likely comparable to a performed under a subset of ecologically relevant conditions that would cause heightened vigilance, such as the presence of predators (Delcourt & Poncin, 2012; Pitcher, 1983). After the recording was completed, length (dorsal mantle length for squid and fork length for fish, both in mm) was determined for each specimen, and averaged per group (Table 2).

### 2.2 Data analysis

#### 2.2.1 Collective organization

Because all measurements were digitized manually from videos, we analyzed a selection of the footage collected sufficient to test hypotheses with statistical power, but manageable enough for data processing feasibility. For in situ footage, the central 2 s of footage was analyzed; for laboratory footage, 1 s every five min was analyzed (the second immediately before each strobe flash). Frames were extracted from selected footage segments at 5.5 frames s^−1^. Thus 11 frames were analyzed per in situ group (*n* = 5 per species) and 25 frames per laboratory group (*n* = 3 per species). Frames were processed in Python using a custom graphical user interface. Two points were manually digitized on all unobscured individuals in each frame (maximum of 10 individuals per in situ frame): one on the head between the eyes, and another behind the dorsal fin for sardine or on the distal mantle tip for squid. The line segment connecting these two points was used for subsequent analyses. Unless otherwise noted, all subsequent analyses were conducted in R version 3.5.2 (R Core Team, 2018), alpha was set to 0.05, and assumptions of statistical tests were checked and met using standard diagnostic tools available in base R or the referenced R packages.

For in situ footage, group organization was quantified in terms of collective alignment using angular deviation (°), a directional organization metric defined as the absolute difference between an individual’s orientation and the average group orientation. For laboratory footage, group organization was additionally quantified in terms of conspecific attraction, which we approximated using nearest neighbor distance (NND, body lengths), a spatial organization metric defined as the distance between an individual’s midpoint and that of its closest groupmate (Herbert‐Read, 2016; Sumpter et al., 2018). NND was not possible to measure from in situ footage because, with only one camera, organization in the vertical plane could not be determined. This same issue was present with laboratory groups, but due to the relatively shallow water depth (0.65 m) and overhead camera location (Figure 2b), the error in NND due to the vertical plane of organization was likely less than that for in situ groups. Moreover, the distance between neighbors in the vertical plane is usually substantially less than in the horizontal plane (Pavlov & Kasumyan, 2000).

To determine angular deviation for in situ groups, the orientation of each individual’s segment was calculated with respect to a fixed vanishing point at the opening of the net (Figure 2c). This allowed us to calculate orientation in the geometric plane of swimming direction, and also to account for discrepancies in apparent orientation due to varying distance from the camera. For laboratory groups, the camera plane was orthogonal with the plane of swimming. Thus, line segment orientation could be determined with respect to default image degree coordinates (Figure 2d). Unlike sardine, squid can swim backward (mantle tip‐first) or forward (head‐first). At sustained fast swimming speeds, like those exhibited in situ, squid generally swim backward. However, at slow swimming speeds, like those exhibited in lab, squid can swim backward or forward. Thus, under laboratory conditions, it was possible that some squid in the group would be oriented ~180° from the rest of their groupmates.

To calculate NND, midpoints were determined for each individual’s line segment (Figure 2d). The distance between nearest neighbor midpoints was divided by average group body length to determine NND in terms of body lengths. Squid line segments (running from head to distal mantle tip) recorded dorsal mantle length, a standard metric of squid length. The entire length of sardine was not marked in digitization because the use of the tail for propulsion precludes using this part of the body to reliably determine general orientation. The sardine line segments, which connected the head to posterior dorsal fin edge—an area of the body which shows minimal flexing, were, therefore, extrapolated to fork length using body proportions derived from scientific illustrations (Whitehead et al., 1988). Fork length was used in standardizing NND to body lengths for sardine.

Our study had repeated measures, as data on groups of each species, in situ and in the laboratory, were collected at multiple time points. Temporal autocorrelation was investigated prior to all analyses; if moderate or strong correlation was found, it was accounted for in the relevant analysis using a first‐order autoregressive process. To compare in situ angular deviation between sardine and squid, we implemented a linear mixed‐effects analysis using the R package “nlme” (Pinheiro et al., 2018). Species (sardine vs. squid) was the fixed effect, and to account for the effect of potential intergroup differences in angular deviation within species on the response, we included group as a random intercept term.

To compare group organization in the laboratory between sardine and squid, while accounting for the effect of potential intergroup differences in organization within species on the response, we used two linear mixed‐effects analyses to, respectively, relate angular deviation and NND to species (fixed effect) with group as a random intercept term. To account for changes in organization that could have resulted from duration in the experimental tank, and how this effect could have been species‐specific, both models initially included time elapsed since experiment start (5, 10, 15, 20, or 25 min), and the interaction between species and time elapsed, as fixed effects. If this interaction was not significant, we reran the model without the interaction. If time elapsed was not significant in the reduced model, the final model did not include this fixed effect.

To compare the angular deviation of each species between environments (*in situ* vs. in laboratory), we, respectively, used two linear mixed‐effects analyses where environment was the fixed effect and group was a random intercept term.

#### 2.2.2 Interaction rules

**FIGURE 3:**
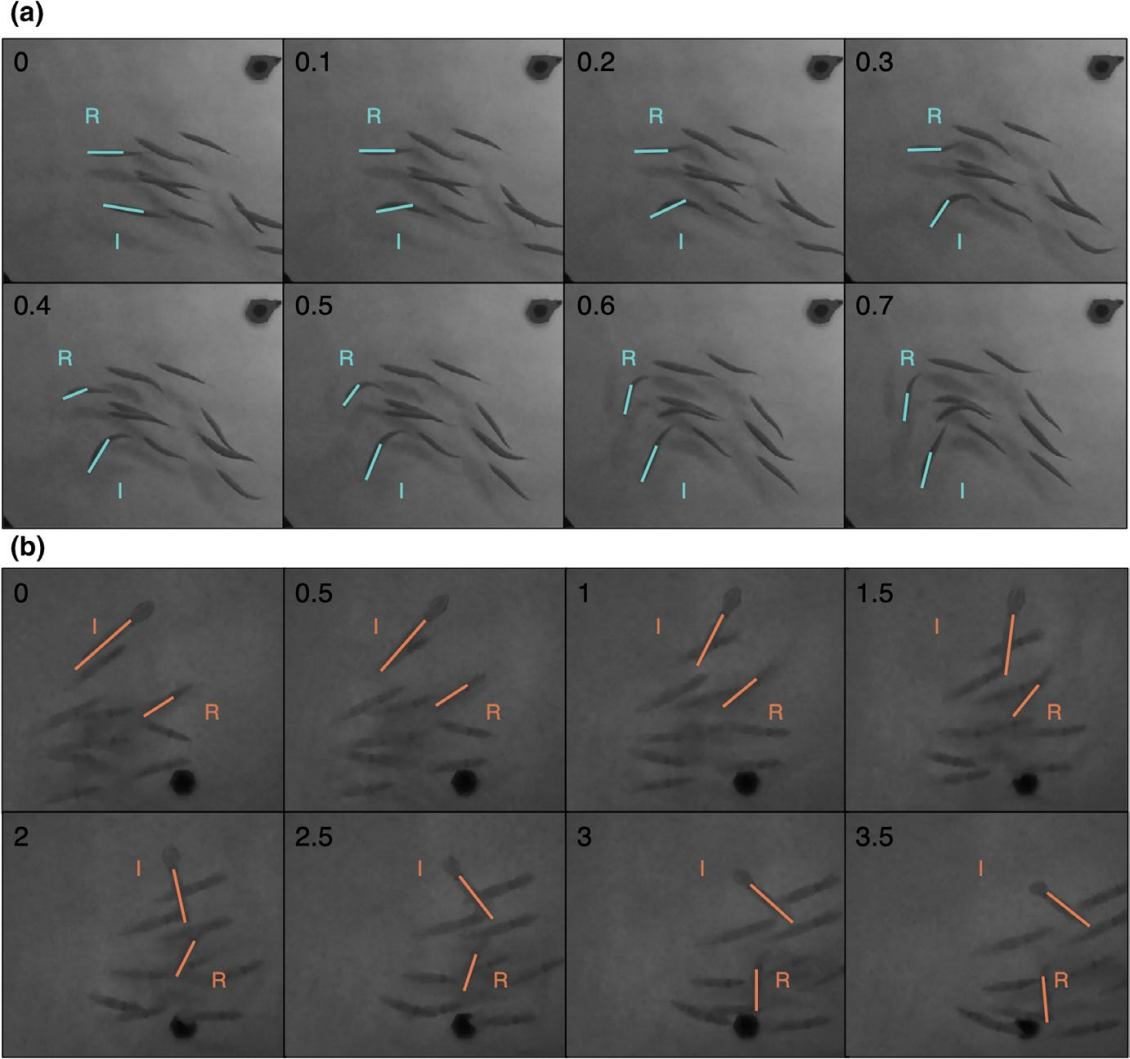
Measuring the latency and extent of responses to spontaneous turns in moving groups of sardine or squid in the laboratory. Illustrative example of a frame analysis for determining the latency and extent of responses to spontaneous turns in (a) sardine and (b) squid. In all, influencers (I) were individuals that executed spontaneous turns and responders (R) were the first individuals to similarly respond to these turns. The orientation of I and R were recorded in each frame using general degree headings (as illustrated in Figure 2d). Time elapsed (s) is indicated in the upper left corner of each frame. The time delay between I and R turns (*τ*, s), and a comparison of R turn rates (angular velocity, ° s^−1^) with I turn rates lagged by *τ*, were, respectively, used to assess latency and extent (see Section 2.2.2). Note that, in (a), the R turn begins between 0.3 and 0.4 s after the I turn, while in (b), the R turn begins between 1 and 1.5 s after the I turn

To investigate how individuals adjust their movements depending on the movements of groupmates, we quantified the latency and extent (i.e., completeness) of responses by each species to spontaneous turns (changes in orientation) of groupmates. We examined the first 10 min of footage collected for two groups of each species in the laboratory. Within species, these groups had similar average length, and between species, this selection included groups with comparable numbers of individuals (Table 2). Within this subset, we analyzed footage segments where one individual executed a spontaneous turn that was quickly followed by similar turns of one or more groupmates (Figure 3). To select these segments in a consistent manner that reduced potential biases, footage was examined multiple times at different playback speeds by the same reviewer. Because sardine exhibited higher instantaneous angular velocity than squid in these footage segments, we extracted frames at 10 frames s^−1^ (thus every 0.1 s) from sardine footage segments and at 2 frames s^−1^ (thus every 0.5 s) from squid footage segments for subsequent analysis. These rates were high enough to quantify angular velocities with reasonable precision for each species, and low enough for data collection feasibility.

Twenty‐one turn‐response footage segments were analyzed for each species (Figure S2A,B). Digitization followed methods described in the previous section for laboratory footage (see Section 2.1.2). However, only two individuals were tracked: the individual that initially executed the spontaneous turn (influencer), and the first individual to respond with a similar turn (responder) (Figure 3). Similar turns were defined as those that qualitatively matched the change in orientation of the influencer. Measurements of degree orientation over time were converted into instantaneous angular velocities (° s^−1^) for subsequent analyses. The time range of turn‐response measurements (i.e., the tracked turn and response duration) was 0.6–2.9 s for sardine and 3.0–6.5 s for squid (Figure S2A,B). The longer tracking durations for squid reflected the lower instantaneous angular velocity than sardine in the footage segments.

Responses to influencer turns occurred after a time lag, or response latency (*τ*), that was potentially species‐specific. To determine *τ* for each turn‐response, we assessed the correlation (Pearson) between responder and influencer instantaneous angular velocities (*ω*_R_ and *ω*_I_, respectively) at different influencer angular velocity time lags (0–0.5 s for sardine, 0–2.5 s for squid) (Figure S2C,D). *τ* for each turn‐response was determined as the time lag (s) with the maximum correlation.

To determine how *τ* was related to species, while accounting for the effect of potential inter‐group differences in responsiveness within species, we used a linear mixed‐effects analysis where species (sardine vs. squid) was a fixed effect and group was a random intercept term. We additionally assessed the potential effects of *ω*_I_, influencer location within the group (edge vs. center), responder location within the group (edge vs. center), and influencer location relative to responder location (lateral vs. anteroposterior) on *τ*. Location within a group was categorized edge or center: an individual was “edge” if it was the outermost individual in any direction (i.e., furthest from the group centroid), otherwise it was “center.” Influencer location relative to the responder was categorized as lateral or anteroposterior: if the influencer was ahead of the responder (also could be behind in squid), its relative location was “anteroposterior;” otherwise its relative location was “lateral.” The initial model included all fixed effects and all possible interactions between fixed effects. If interactions were not significant, we reran the model without interactions. If fixed effects (aside from species) in the subsequent model were not significant, we removed these terms to create a final model that included species, all other significant fixed effects (or nonsignificant fixed effects that were a part of significant interactions), and group as a random intercept.

To assess the extent of responses to influencer turns, we calculated the difference between average *ω*_R_ and average *ω*_I+_ *_τ_* (average influencer angular velocity lagged by *τ*), relative to average *ω*_I+_ *_τ_*, for each turn‐response. Thus, a response extent of 0 would indicate that, on average *ω*_R_ = *ω*_I+_ *_τ_*, while a positive or negative response extent would indicate that *ω*_R_ > *ω*_I+_ *_τ_* or *ω*_R_ < *ω*_I+_ *_τ_*, respectively. In other words, a response extent of 0, >0, or <0 would indicate that the responder matched the movement of the influencer, exceeded the movement of the influencer, or was less than the movement of the influencer, respectively.

To determine how response extent was related to species, while accounting for the effect of potential intergroup differences in responsiveness within species, we used a linear mixed‐effects analysis where species (sardine vs. squid) was a fixed effect and group was a random intercept term. We additionally included the same fixed effects as with the model for *τ* and followed the same modeling procedure previously described for *τ*.

## 3 RESULTS

### 3.1 Collective organization

**FIGURE 4:**
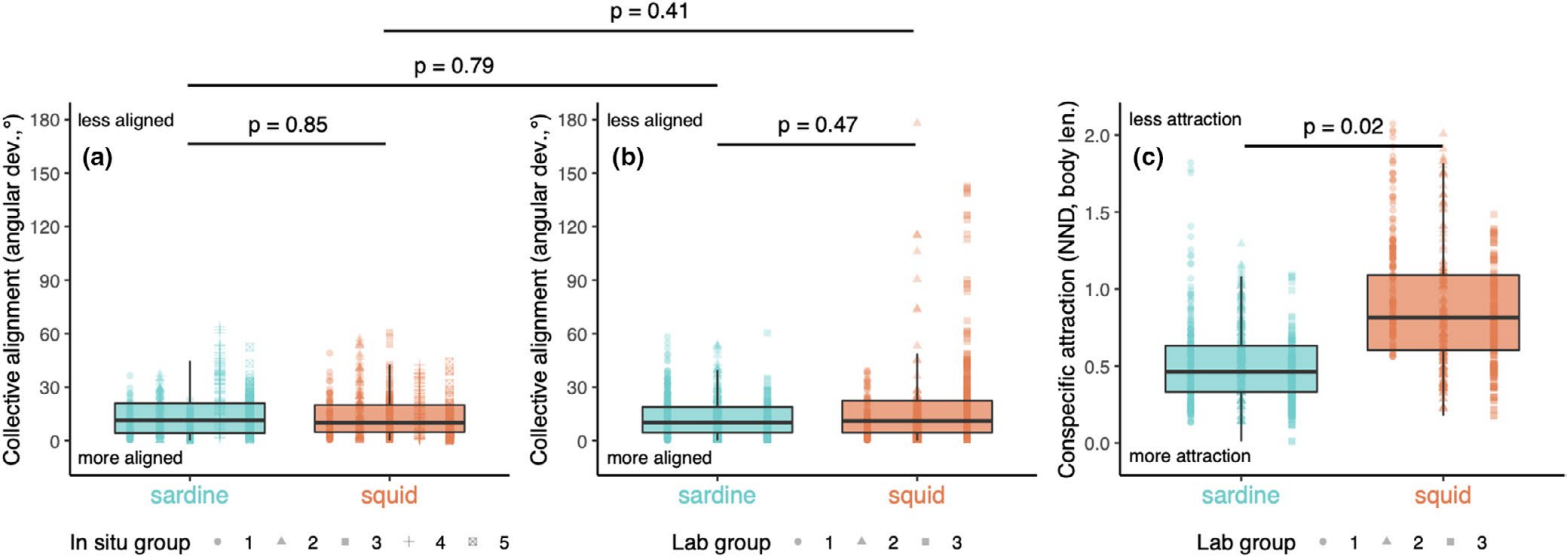
Comparable collective alignment but not conspecific attraction of individuals within moving sardine or squid groups. (a) Alignment, as measured by angular deviation, or the difference between an animal’s orientation and the average group orientation, of 5 groups of each species in situ (*n* = 548 for sardine and 550 for squid). See Table 1 for the total number of individuals in each in situ group; a maximum of 10 unobscured individuals were measured in each analyzed frame, or time point (see Section 2.2). Dark horizontal lines are the median value, with boxes and vertical lines the inner and outer quartiles, respectively. Points are raw measurements, shaped by group and colored by species. Points that fall beyond vertical lines are outliers. (b) Alignment of 3 groups of each species in the laboratory (*n* = 704 for sardine and 562 for squid), with lines, boxes, and points indicating the same attributes as (a). (c) Attraction, as measured by nearest neighbor distance (NND), or the distance between the lengthwise midpoint of an animal and that of its closest groupmate, of 3 groups of each species in the laboratory (*n* = 704 for sardine and 562 for squid). Lines, boxes, and points indicate the same attributes as (a). See Table 2 for the total number of individuals in each laboratory group; all unobscured individuals were measured in each analyzed frame, or time point (see Section 2.2). Horizontal lines above boxplots indicate the significance of the respective difference between species (sardine vs. squid) or environmental context (in situ vs. laboratory) as determined by linear mixed‐effects analyses (see Section 3.1 and Table S1). In (c), time elapsed in experiment had a significant but small effect on the nearest neighbor distance for both species: over the 30 min experiment, spacing increased by 9% of the average group body length

Squid groups had comparable collective alignment to sardine groups. Angular deviation, an alignment (directional organization) metric defined as the difference between individual orientation and average group orientation, was not different between sardine and squid measured in situ (DF = 8, *t* = −0.19, *p* = .85; Figure 4a; Table S1A) or in the laboratory (DF = 4, *t* = 0.79, *p* = .47; Figure 4b; Table S1B). In the latter comparison, there was no significant effect of time elapsed during the experiment on angular deviation. On average (±SE), in situ angular deviation was 13.6 ± 2.85° for squid and 14.4 ± 2.85° for sardine; in the laboratory, the angular deviation was 18.1 ± 4.52° for squid and 13.0 ± 4.51° for sardine. Angular deviation was not different between environmental contexts (in situ vs. in laboratory) in sardine (DF = 6, *t* = −0.27, *p* = .79; Figure 4a,b; Table S1C) nor in squid (DF = 6, *t* = 0.89, *p* = .41; Figure 4a,b; Table S1D). Intergroup differences in alignment had no consistent qualitative association with average length or group size in either species (see Results S1 and Figure S1).

Squid groups had lower conspecific attraction than sardine groups in the laboratory. Nearest neighbor distance (NND), an attraction‐related (spatial organization) metric defined as the distance between an individual’s lengthwise midpoint and that of the closest groupmate, was greater in squid than in sardine by (average ± SE) 0.38 ± 0.11 body lengths (DF = 4, *t* = 3.62, *p* = .02; Figure 4c; Table S1E). In this comparison, there was a significant effect of time elapsed during experiment on NND for both species: on average (±SE), NND increased by 0.003 ± 0.001 body lengths min^−1^ during the 30 min experiment (DF = 1259, *t* = 2.18, *p* = .03). At the middle of experiments (15 min elapsed), the NND of sardine was 0.50 ± 0.07 body lengths, and the NND of squid was 0.88 ± 0.08 body lengths. Qualitatively, individuals in larger sardine groups were spaced farther apart, while larger (and longer) squid groups had individuals that were spaced closer together (see Results S1 and Figure S1).

### 3.2 Interaction rules

Spontaneous turns and responding turns were much faster in sardine than squid. Sardine influencers, or individuals that executed obvious turns, and responders, or the first individuals to similarly respond to these turns, had a higher average (±1 SD) maximum instantaneous angular velocity (402 ± 106 and 394 ± 106° s^−1^, respectively) than squid influencers or responders (30.3 ± 11.0 and 30.9 ± 11.0° s^−1^, respectively) (Figure S2A,B).

**FIGURE 5:**
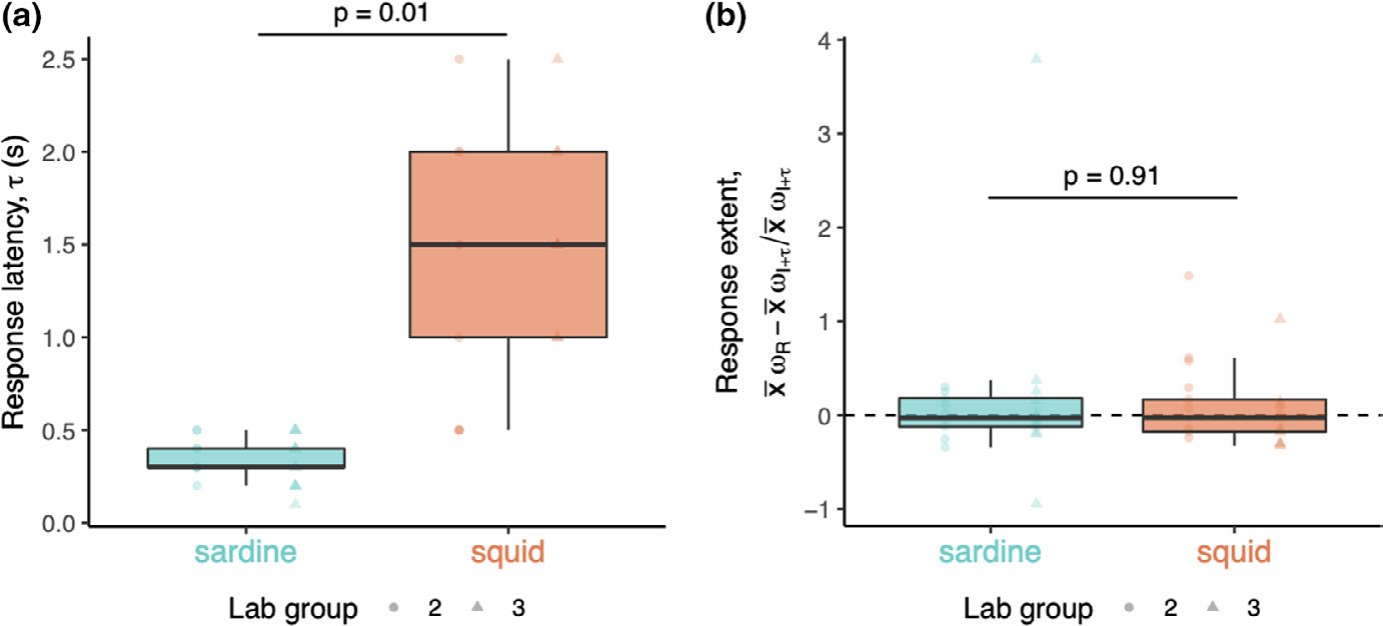
Different latency but comparable extent of responses to turns in moving groups of sardine or squid. (a) Response latency, *τ*, or the time lag of maximum correlation between responder and influencer angular velocity (*ω*_R_ and *ω*_I_, respectively) (see Figure S2C,D), measured in two groups of each species in the laboratory (*n* = 21 in each species). Dark horizontal lines are the median value, with boxes and vertical lines the inner and outer quartiles, respectively. Points are raw measurements, shaped by group and colored by species. Points that fall beyond vertical lines are outliers. (b) Response extent, or the difference between average *ω*_R_ and average *ω*_I+_ *_τ_* (average influencer angular velocity lagged by *τ*), relative to average *ω*_I+_ *_τ_*, measured in two groups of each species in the laboratory (*n* = 21 in each species). Lines, boxes, and points indicate the same attributes as (a). The horizontal dashed line is at *y* = 0, or average *ω*_R_ = average *ω*_I+_ *_τ_*; values above this line indicate average *ω*_R_ > average *ω*_I+_ *_τ_*, while those below indicate average *ω*_R_ < average *ω*_I+_ *_τ_*. Solid horizontal lines above boxplots indicate the significance of the respective difference between species (sardine vs. squid) as determined by linear mixed‐effects analyses (see Table S1F,G)

Squid also responded more slowly to the spontaneous turns of groupmates than sardine. Response latency (*τ*), or the time lag of maximum correlation between responder and influencer angular velocities (*ω*_R_ and *ω*_I_, respectively; Figure S2C,D) measured in the laboratory, was greater in squid than in sardine (DF = 2, *t* = 8.76, *p* = .01; Figure 5a; Table S1F). In this comparison, there was no significant effect of *ω*_I_, influencer location within the group (edge vs. center), responder location within the group (edge vs. center), or influencer location relative to responder location (lateral vs. anteroposterior) on *τ*. On average (±SE), *τ* was 0.34 ± 0.09 s in sardine and 1.48 ± 0.09 s in squid. Group *τ* intercept residuals were negligible (Table S1F).

In both species, the spontaneous turn rate was matched by the responding turn rate. Response extent, or the difference between average *ω*_R_ and average *ω*_I+_ *_τ_* (average influencer angular velocity lagged by *τ*), relative to average *ω*_I+_ *_τ_*, was not different between sardine and squid measured in the laboratory (DF = 2, *t* = −0.13, *p* = .91; Figure 5b; Table S1G). Like *τ*, there was no significant effect of *ω*_I_, influencer location within the group (edge vs. center), responder location within the group (edge vs. center), or influencer location relative to responder location (lateral vs. anteroposterior) on response extent. On average, response extent was 0.15 ± 0.15 in sardine and 0.12 ± 0.15 in squid, which was not significantly different from 0 (DF = 38, *t* = 0.96, *p* = .34). Also, like *τ*, group response extent intercept residuals were negligible (Table S1G).

## 4 DISCUSSION

### 4.1 Collective organization

Using standardized methods, we assessed the convergence in collective behavior between two ecologically similar, competing marine species: California market squid, *D*. *opalescens*, and Pacific sardine, *S*. *sagax*. We found that groups of each had comparably high collective alignment (low angular deviation) during collective motion, both *in situ* and in the laboratory. Individuals of both species tended to orient 13–14° from the group’s average heading in either context (Figure 4a,b). These values are within the range of those previously reported for clupeid fishes (generally within 5–25°, Pavlov & Kasumyan, 2000), and for loliginid squid: 9.1° was the minimum average angular deviation between adult *D*. *opalescens* in the laboratory (Hurley, 1978), and 42.7° was the average maximum nearest neighbor angle between hatchling oval squid (*Sepioteuthis lessoniana*) in the laboratory (Sugimoto & Ikeda, 2012).

Common alignment facilitates group‐level benefits, including information transfer between groupmates (MacGregor et al., 2020; Sumpter et al., 2018). In collectively moving groups, changes in individual orientation can signal ecologically important information—for example, the location of a new nest site in honeybee, *Apis mellifera* (Schultz et al., 2008) or the approach of a predator in northern anchovy, *Engraulis mordax* (Cade et al., 2020). Moreover, both social fish and squid often have pigmentation patterning along their bodies that would be most readily perceived when individuals are aligned (Hanlon & Messenger, 2018; Pavlov & Kasumyan, 2000). Pacific sardine has several spots running along its lateral surface (Figure 1b), while California market squid produces a large repertoire of stereotyped pigmentation patterns involving stripes and splotches (Hunt et al., 2000; Zeidberg, 2004). Pigmentation patterning in fish could have signaling value during collective movements, determine group behaviors, facilitate organization, and reinforce unification (Pavlov & Kasumyan, 2000), and the same may be true of social squid (Burford & Robison, 2020; Hanlon & Messenger, 2018). High alignment in both squid and sardine could therefore indicate behavioral convergence in collective motion in these species due to the functional importance of information transfer.

We found that conspecific attraction was high in both species (i.e., low NND) in the laboratory, but that squid were positioned 83% farther from their nearest neighbors than sardine (Figure 4c). It is important to note that measured body length in squid did not include the arms (as is standard practice), but this is unlikely to have meaningfully affected our reported NND difference between species. Our values for NND were relatively low in both species: on average (±SE) NND was 0.88 ± 0.07 body lengths in squid and 0.50 ± 0.07 body lengths in sardine at the midpoint of experiments (i.e., 15 min elapsed). Previously measured values of attraction in squid groups range from a minimum spacing of 0.6 body lengths in wild groups of Humboldt squid, *Dosidicus gigas* (minimum interindividual distance [25 cm] in terms of the average of the reported size range [4–84.5 cm], Benoit‐Bird & Gilly, 2012), to a NND of 5.3 body lengths in hatchling oval squid in the laboratory (Sugimoto & Ikeda, 2012). In the wild, NND within large fish schools is typically 1.5–3 body lengths, but under laboratory conditions, separation distance can be as small as 0.1–0.6 body lengths (Pavlov & Kasumyan, 2000). Available data suggest the same is true for squids; in the laboratory, NND in groups of oval squid was 1.8 body lengths, while in the wild NND in groups could be as high as 3 body lengths depending on group shape (Sugimoto & Ikeda, 2012; Sugimoto et al., 2013). We found that attraction decreased (i.e., NND increased) through the 30‐min experiments at a rate of 0.003 ± 0.001 body lengths min^−1^ for both California market squid and Pacific sardine (Table S1E). Although we do not know how attraction changed during the 1‐hour acclimation period, there is little evidence to suggest it would be different between species.

The degree of spacing between individuals is often related to group‐level benefits, including energetic savings. The differences we observed in relative spacing between squid and sardine groups could stem from differences in their locomotory modes. In schooling fish, precise spacing often allows individuals to take advantage of hydrodynamic effects created by other swimming groupmates that make swimming less energetically expensive. For instance, individuals can exploit zones of higher pressure in front of other swimming groupmates (i.e., “bow‐riding”), or pressure gradient zones diagonally or behind other swimming groupmates (i.e., “drafting”) created by vortex shedding (Liao et al., 2003; Marras et al., 2015; Pavlov & Kasumyan, 2000). While the vortices produced by squid jet propulsion (Bartol et al., 2016) and the wake structures produced by squid fin undulation (Stewart et al., 2010) have been described, potential hydrodynamic advantages relevant to squid grouping have not been investigated. The difference in conspecific attraction between sardine and squid we observed could reflect differences in positioning required to take advantage of fluid disturbances. Alternatively, lower attraction in squid compared to sardine may reflect differences in maneuverability between these species, as has been suggested for different fish species (Partridge et al., 1980).

In addition to energy savings, conspecific attraction may also be related to defense from predator attacks. In response to predators, attraction within groups of prey tends to increase (thus NND decreases) as individuals seek central positions within the group (Krause, 1993), and this increases group density. Predators that target individuals within groups more frequently attack the denser regions of groups because they are more conspicuous (Ioannou et al., 2009; Parrish, 1989). However, the accuracy of their attacks is lower in denser regions of prey groups due to the confusion effect of nearest neighbors surrounding the target (Ioannou et al., 2009). Many upper trophic level species that prey on sardine and squid, including birds, fish, and marine mammals (Morejohn et al., 1978), target individuals within groups. Thus, differences in attraction between sardine and squid groups could reflect defenses against such predators at different spatial scales (Ioannou et al., 2009): lower attraction in squid could make them less obvious to searching predators, while higher attraction in sardine could better protect individuals once predators are attacking.

Unlike predators that target individuals within groups, lunge feeders, which engulf and filter huge water volumes containing prey aggregations, benefit from higher prey densities (Goldbogen et al., 2019). Small schooling fish, including sardine, are often the target of lunge feeding whales in the California Current System (CCS). For instance, the humpback whale (*Megaptera novaeangliae*), which migrates to the CCS to feed, is an opportunistic predator that has been shown from historical records to primarily feed on sardine and krill (Clapham et al., 1997). More recently, northern anchovy (*Engraulis mordax*) has replaced sardine in whale diets since the sardine populations in the CCS crashed in the 1950’s (Fleming et al., 2016). Small grouping squid, such as California market squid, seem to be rarely targeted by lunge feeding whales even though they are present in comparable or greater numbers than sardine or anchovy (Harding et al., 2021; Sakuma et al., 2016). Historical records indicate squid (most likely California market squid) have been found in humpback whale stomach contents in the CCS, but rarely constitute a meaningful proportion (Clapham et al., 1997). This is peculiar because, in addition to grouping in large numbers throughout their nektonic life, California market squid form massive spawning aggregations (Zeidberg, 2013). Both types of aggregations, which are targeted by a host of other upper trophic level species in the CCS (Morejohn et al., 1978), can overlap in time and space with schools of anchovy or sardine (Sakuma et al., 2016). Similar results—aggregating squid rarely being present in the diet of lunge feeding whales—have also been reported from the western north Pacific (Nemoto, 1970), so this phenomenon may not be unique to the CCS. There could be energetic reasons that lunge feeding whales do not commonly target squid, including their lower lipid content and higher protein content compared with fish (Burford et al., 2022), as protein is more energetically expensive to metabolize than lipid (Schmidt‐Nielsen, 1997). Our finding that individuals within groups of squid were spaced 83% farther apart than sardine suggests that squid group density could be another factor making this taxon less energetically worthwhile for lunge feeders to target than fish. Cade et al. (2020) found that the energetic benefits for lunge feeding whales targeting schooling fish were extremely sensitive to attack timing, as well as aspects of the prey’s behavior such as escape response latency and packing density (i.e., NND). Thus, very small differences in these parameters can be the difference between energetically beneficial lunges and wasted ones (Cade et al., 2020).

In this study, we observed that both squid and sardine were highly collectively aligned. We also observed that conspecific attraction was relatively high (i.e., low NND) compared with previous field studies (Pavlov & Kasumyan, 2000). Our results, however, were recorded within the confines of a large trawl net and a tank in the laboratory. Heightened vigilance, including that which could be exhibited in an environment with threats such as predators, likely affected observed behavior (Delcourt & Poncin, 2012; Pavlov & Kasumyan, 2000; Pitcher, 1983; Schaerf et al., 2017). Thus, our data probably represent how these collective behaviors are performed only under a subset of ecologically relevant conditions, such as under immediate threat from a predator. Yet under these conditions, squid and sardine show convergent, highly comparable collective behaviors.

The goal of this study was to focus on between‐species variation while keeping the environment consistent. To accomplish this, we used standardized observation methods, and statistical models that explicitly considered intergroup variation within species. Such differences could potentially be due to differences in body size or group size, but no striking or consistent associations between these metrics and alignment or attraction were recorded in either sardine or squid (see Results S1 and Figure S1). As individuals grow, age, and learn, interaction strengths that affect group cohesion generally increase (Herbert‐Read, 2016; Romenskyy et al., 2017). In contrast, increases in group size generally do not affect how individuals interact (Katz et al., 2011), but it can increase the frequency of interactions, affecting group cohesion.

### 4.2 Interaction rules

Animal groups achieve coordinated motion using interaction rules, or consistent modes by which individuals adjust their movements depending on groupmates’ information (Herbert‐Read, 2016). We investigated a potential interaction rule that could explain the comparably high alignment of sardine or squid groups during collective motion—the responsiveness of individuals to spontaneous turns of groupmates in laboratory. In both species, individuals responded to spontaneous turns by executing similar turns—that is, the relative difference between the angular velocity (*ω*) of spontaneous turners and responders was negligible (Figure 5b). However, the latency of responses to turns (*τ*) was considerably longer in squid versus sardine—on average, squid responses were delayed by 1.5 s while sardine took an average of 0.3 s to respond.

This difference does not reflect physiological constraints on response rates to visual stimuli in squid. When exposed to camera‐strobe flashes under temperature and oxygen conditions like those in our experiments, California market squid show a latency of 50–75 ms for muscular activity of the mantle that produces the escape jet, and this activity is preceded by head retraction (Otis & Gilly, 1990). Of course, these are largely reflexive actions that involve minimal processing by the brain and probably no conscious decision‐making. Differences in *τ* between sardine and squid could reflect differences in each species’ interaction rules, and any relevant decision‐making, as opposed to differences in their capacity to respond to visual stimuli reflexively.

During the turn‐response instances that we examined, sardine had a much higher average maximum instantaneous angular velocity (394–402° s^−1^) than squid (30.3–30.9° s^−1^), and this could have affected *τ*. However, influencer angular velocity (*ω*_I_) was not a significant fixed effect in models of *τ* (see Section 3.2). The brief squid (*Lolliguncula brevis*), a close relative of the California market squid, exhibits an average (±SE) maximum instantaneous angular velocity of 268 ± 32.9° s^−1^ spontaneously (Jastrebsky et al., 2016); this value is even higher when attacking fish prey (approach = 303 ± 50.7° s^−1^, recoil [the period from prey contact to wrapping the prey in the arms] = 444 ± 55.6° s^−1^; Jastrebsky et al., 2017), values more similar to those we observed in sardine than in market squid. Observations of rapid turns in the California market squid in situ (Hunt et al., 2000) and in the laboratory suggest this species is, however, capable of comparable turning rates to brief squid and sardine. Thus, instead of directly related to turning rates, the difference in *τ* between sardine and squid probably reflects unique interaction rules that account for differences in locomotion (O’Dor & Webber, 1986).

Sardine are obligate forward swimmers; in contrast, at slow speeds, squid can swim backwards and forwards equally well using a mixture of jet propulsion and fin undulation (Bartol et al., 2008). In some cases, we observed that individuals within squid groups would rotate 180 ° from the group heading and continue to hold position within the group without the rest of the group responding (see upper outer quartile in Figure 4b). Thus, the high latency of alignment responses in squid may reflect interaction rules that allow individuals to engage in social behaviors involving orientation reversals without causing similar shifts throughout the group. Evidence suggests social responsiveness is a context‐dependent trait (Herbert‐Read, 2016; Schaerf et al., 2017). At fast swimming speeds when squid use jet propulsion to swim mantle tip‐first, response latency to turns is likely lower than that we observed in the laboratory.

While some theoretical models of collective motion include alignment terms (e.g., Couzin et al., 2002), empirical evidence suggests that animal groups can achieve alignment without explicitly matching the orientation of groupmates. Alignment instead results from selective repulsion and attraction to individuals in lead positions at small group sizes, or nearest neighbors at larger group sizes (Katz et al., 2011). This evidence comes from small (~5 cm) golden shiner (*Notemigonus crysoleucas*), a fish that grows to 30 cm in the wild. Smaller individuals within grouping species often show weaker interindividual attraction and do not adhere as strictly to the internal structures of groups compared with larger individuals within species (Hurley, 1978; Mather & O’Dor, 1984; Pavlov & Kasumyan, 2000; Romenskyy et al., 2017; Sugimoto & Ikeda, 2012). Therefore, interaction rules within species may change as individuals grow, learn, and age (Herbert‐Read, 2016). Thus, as our results suggest, it is plausible that adult sardine and squid explicitly match the orientation of individuals in lead positions or nearest neighbors. This interaction rule may be partly responsible for the high alignment of groups of each species during collective motion.

## CONFLICT OF INTEREST

The authors have no conflict of interest to declare.

## AUTHOR CONTRIBUTIONS

**Benjamin P. Burford:** Conceptualization (equal); Data curation (lead); Formal analysis (lead); Methodology (equal); Validation (lead); Visualization (lead); Writing – original draft (lead); Writing – review & editing (lead). **R. Russell Williams:** Conceptualization (equal); Formal analysis (equal); Software (lead); Writing – review & editing (equal). **Nicholas J. Demetras:** Conceptualization (equal); Data curation (equal); Methodology (equal); Writing – review & editing (equal). **Nicholas Carey:** Conceptualization (equal); Methodology (supporting); Resources (equal); Writing – review & editing (equal). **Jeremy Goldbogen:** Funding acquisition (equal); Resources (equal); Supervision (equal); Writing – review & editing (equal). **William F. Gilly:** Funding acquisition (equal); Resources (equal); Supervision (equal); Writing – review & editing (equal). **Jeffrey Harding:** Conceptualization (equal); Data curation (equal); Methodology (equal); Resources (equal); Supervision (equal); Writing – review & editing (equal). **Mark W. Denny:** Funding acquisition (equal); Resources (equal); Supervision (equal); Writing – review & editing (equal).

## Supporting information

Supplementary Material

Video S1

Video S2

Video S3

Video S4

## Data Availability

The data generated in this study have been deposited in the Dryad Digital Repository (https://doi.org/10.5061/dryad.q573n5tm0; Burford, 2022).
