# Supplementary material for: The limits of convergence in the collective behavior of competing marine taxa
Source: Ecol Evol. 2022 Mar 22;12(3):e8747. doi: 10.1002/ece3.8747 (PMC8939367; doi:10.1002/ece3.8747)
Supplement: Supplementary file 1 — Supplementary Material [file ECE3-12-e8747-s005.pdf]

Ecology and Evolution
Open Access

Supporting information for

The limits of convergence in the collective behavior of competing marine taxa

Benjamin P. Burford[1,2]*, R. Russell Williams[1], Nicholas J. Demetras[2], Nicholas Carey[1,3], Jeremy Goldbogen[1], William F. Gilly[1], Jeffrey Harding[4], Mark W. Denny[1]

1 Hopkins Marine Station of Stanford University, Pacific Grove, CA, 93950, USA

2 University of California Santa Cruz, Institute of Marine Sciences, affiliated with the National Oceanic and Atmospheric Administration, National Marine Fisheries Service, Southwest Fisheries Science Center, Santa Cruz, CA, 95060, USA

3 Marine Scotland Science, Aberdeen, United Kingdom

4 National Oceanic and Atmospheric Administration, National Marine Fisheries Service, Southwest Fisheries Science Center, Santa Cruz, CA, 95060, USA

*Corresponding author: bburford@ucsc.edu

This PDF file includes

Results S1

Figures S1 and S2

Table S1

And captions for Video S1-S4 (separate files)

Results S1

Inter-group differences in alignment had no consistent qualitative association with average length or group size in either species. Group angular deviation intercept residuals, or the difference between a group's angular deviation and the species' average angular deviation as estimated from linear mixed-effects analyses, ranged from -4.70 to 3.15 ° in squid and -8.50 to 12.6 ° in sardine *in situ*, and from -7.88 to 11.9 ° in squid and -2.88 to 2.35 ° in sardine in lab (Figure S1; Table S1). *In situ* values had no clear association with group size or average length in sardine (Figure S1A). However, there was minimal variation in the average length of sardine groups measured *in situ* (116-119 mm; Table 1). In contrast, squid groups with smaller average length tended to have higher angular deviation (less aligned) than groups of larger average length (Figure S1A). However, smaller-sized groups were all captured in the same trawl, and thus average length could not be precisely estimated at the group level (Table 1). In groups of sardine measured in lab, the smallest group of 9 individuals had slightly lower angular deviation (more aligned) than the larger two groups, which each contained 11 individuals each (Figure S1B). Similar to *in situ* data, there was minimal variation in average length between sardine groups measured in lab (192-195 mm, Table 2). In contrast to *in situ* data, there was no clear association between average length and angular deviation in squid groups measured in lab (Figure S1B). However, all squid used in lab were of larger average length (117-137 mm, Table 2) than *in situ* groups (73-80 mm, Table 1). As with *in situ* groups, there was no clear association between group size and angular deviation in lab squid groups. To summarize, groups of longer squid were more aligned than groups of shorter squid *in situ*, while smaller sardine groups were more aligned than larger sardine groups in lab.

Inter-group differences in attraction had opposite qualitative associations with group size in each species. The NND intercept residuals, or the difference between a group's NND and the species' average NND as estimated from linear mixed-effects analyses, ranged from -0.11 to 0.18 body lengths in squid and -0.08 to 0.01 body lengths in sardine. NND intercept residuals were lowest (most attraction) in the smallest group of sardine and highest (least attraction) in the smallest group of squid (Figure S1C). The smallest group of squid was also that with the shortest average length (117 mm, Table 2) of squid groups measured in lab. In other words, individuals in larger sardine groups were spaced farther apart, while larger (and longer) squid groups had individuals that were spaced closer together.

Within squid, inter-group variation in collective alignment and conspecific attraction generally agreed with previous lab research findings of squid (Hurley 1978, Webber & O'Dor 1982, Sugimoto & Ikeda 2012). Qualitatively, alignment and attraction were greater in groups with larger body size or groups with more individuals (Figure S1A, C). However, alignment in lab had no discernable association with body size or group size (Figure S1B). In contrast, inter-group variation in sardine alignment and attraction qualitatively showed no association (Figure S1A) or the opposite association (Figure S1B, C). However, it is important to note that sardine body size had less between-group variation that that of squid, and the same is true for between-group variation in group size in lab. *In situ* evidence for squid suggests spacing, as measured by average inter-individual distance, increases with body size (Benoit-Bird & Gilly 2012, Benoit-Bird et al. 2017), which is the opposite trend reported from lab studies. In any case, inter-group differences in organization within our study species were considered when statistically comparing collective behaviors between species.

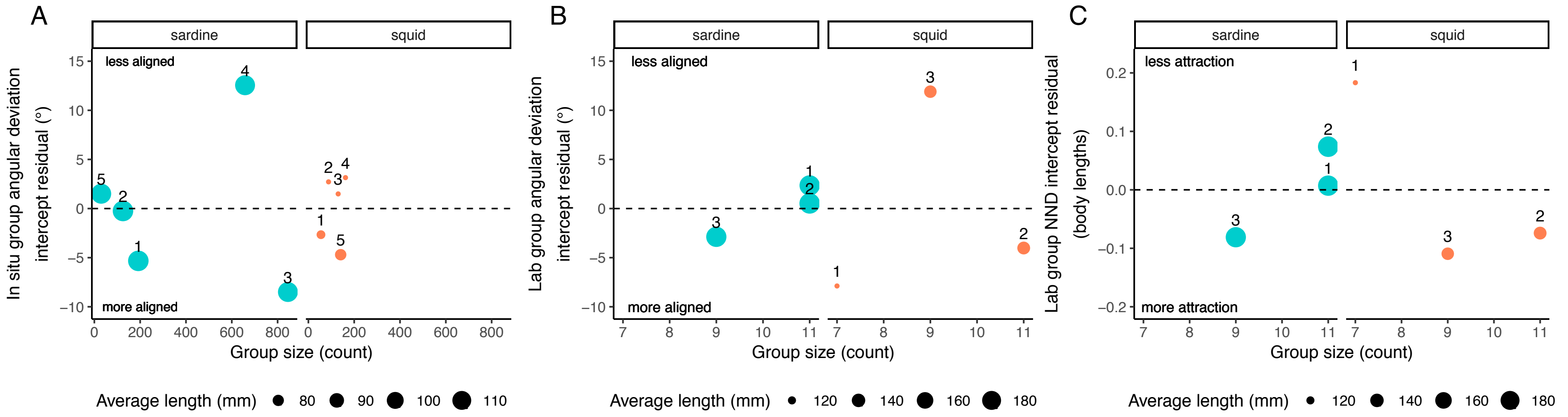

Figure S1 Group-specific effects on species' collective organization were not consistently related to average length or group size. Our study had repeated measures, as data on groups of each species were collected at multiple time points. We therefore used a random intercept term in regressions comparing angular deviation (collective alignment) or nearest neighbor distance (NND, conspecific attraction) between species (sardine vs. squid) or environmental contexts (*in situ* vs. lab) that accounted for the potentially unique organization of each group. A) *In situ* angular deviation intercept residual, or the difference between an *in situ* group's angular deviation and the species' angular deviation, as estimated from linear mixed-effects analyses (see Table S1A). See Table 1 for the total number of individuals in each *in situ* group; a maximum of 10 unobscured individuals were measured in each analyzed frame, or time point (see Materials and methods, Data analysis). B) Angular deviation intercept residual for lab groups of each species (see Table S1B). C) NND intercept residual for lab groups of each species (see Table S1E). See Table 2 for the total number of individuals in each Lab group; all unobscured individuals were measured in each analyzed frame, or time point (see Materials and methods, Data analysis). In (A-C) group intercept residual points are sized according to the average length of each group, labeled with the group number (same numbers as in Figure 3), and plotted with respect to the total number of individuals within that group (x-axis). The

horizontal dashed lines at y = 0 indicate the respective species intercept (average) value, as estimated from linear mixed-effects analyses (see Table S1); points above or below this line represent groups with values > or < the species value, respectively.

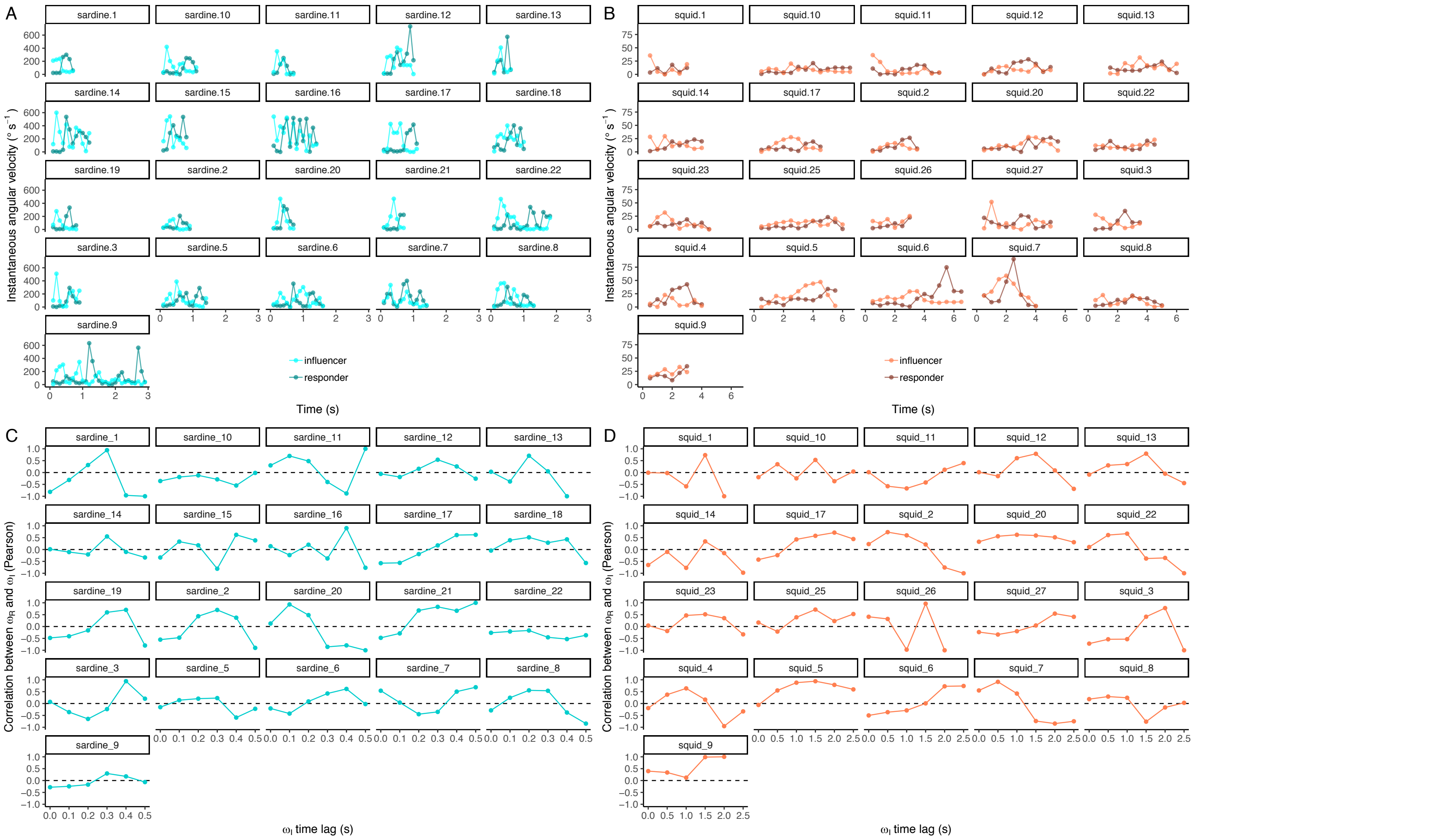

Figure S2 Responder and influencer instantaneous angular velocities, and their correlation at different time lags, in moving groups of sardine and squid measured in lab.

Influencers were individuals that executed spontaneous turns and responders were the first individuals to similarly respond to these turns (see Figure 2). Responder and influencer angular velocity (**ω_R** and **ω_I**, respectively) over time for (A) sardine and (B) squid.

Correlation between $\boldsymbol{\omega}_R$ and $\boldsymbol{\omega}_I$ at (C) 0-0.5 s time lags in sardine and (D) 0-2.5 s time lags in squid. For reference, horizontal lines were placed at y (correlation) = 0; response latency, $\boldsymbol{\tau}$, was determined as the time lag (s) of maximum correlation. Above each plot in (A-D), the unique turn examined (_#) follows the species name (n = 21 in each species).

A) Species comparison of in situ alignment (°), related to Figure 3A & Figure S1A

Effect	Term	Estimate	SE	DF	t-value	p-value
Fixed	Intercept (sardine)	14.38	2.85	1088	5.04	< 0.001
	Squid intercept diff.	-0.80	4.03	8	-0.20	0.85
Random	Sardine in situ group 1 intercept diff.	-5.32				
	Sardine in situ group 2 intercept diff.	-0.25				
	Sardine in situ group 3 intercept diff.	-8.50				
	Sardine in situ group 4 intercept diff.	12.56				
	Sardine in situ group 5 intercept diff.	1.51				
	Squid in situ group 1 intercept diff.	-2.66				
	Squid in situ group 2 intercept diff.	2.72				
	Squid in situ group 3 intercept diff.	1.49				
	Squid in situ group 4 intercept diff.	3.15				
	Squid in situ group 5 intercept diff.	-4.70				

B) Species comparison of lab alignment (°), related to Figure 3B & Figure S1B

Effect	Term	Estimate	SE	DF	t-value	p-value
Fixed	Intercept (sardine)	13.01	4.51	1260	2.88	0.004
	Squid intercept diff.	5.05	6.39	4	0.79	0.47
Random	Sardine lab group 1 intercept diff.	2.35				
	Sardine lab group 2 intercept diff.	0.53				
	Sardine lab group 3 intercept diff.	-2.88				
	Squid lab group 1 intercept diff.	-7.88				
	Squid lab group 2 intercept diff.	-4.02				
	Squid lab group 3 intercept diff.	11.90				

C) Environment comparison of sardine alignment (°), related to Figure 3A, B & Figure S1A, B

Effect	Term	Estimate	SE	DF	t-value	p-value
Fixed	Intercept (in situ)	14.38	3.10	1244	4.64	< 0.001
	Lab intercept diff.	-1.37	5.04	6	-0.27	0.79
Random	Sardine in situ group 1 intercept diff.	-5.32				
	Sardine in situ group 2 intercept diff.	-0.25				
	Sardine in situ group 3 intercept diff.	-8.50				
	Sardine in situ group 4 intercept diff.	12.56				
	Sardine in situ group 5 intercept diff.	1.51				
	Sardine lab group 1 intercept diff.	2.35				
	Sardine lab group 2 intercept diff.	0.53				
	Sardine lab group 3 intercept diff.	-2.88				

D) Environment comparison of squid alignment (°), related to Figure 3A, B & Figure S1A, B

Effect	Term	Estimate	SE	DF	t-value	p-value
Fixed	Intercept (in situ)	13.58	3.12	1104	4.36	0
	Lab intercept diff.	4.49	5.05	6	0.89	0.41
Random	Squid in situ group 1 intercept diff.	-2.66				
	Squid in situ group 2 intercept diff.	2.72				
	Squid in situ group 3 intercept diff.	1.49				
	Squid in situ group 4 intercept diff.	3.15				
	Squid in situ group 5 intercept diff.	-4.70				
	Squid lab group 1 intercept diff.	-7.88				
	Squid lab group 2 intercept diff.	-4.02				
	Squid lab group 3 intercept diff.	11.90				

E) Species comparison of lab attraction (NND, body lengths), related to Figure 3C & Figure S1C

Effect	Term	Estimate	SE	DF	t-value	p-value
Fixed	Intercept (sardine)	0.46	0.08	1259	6.04	< 0.001
	Squid intercept diff.	0.38	0.11	4	3.62	0.02
	Time elapsed intercept diff. (per min)	0.003	0.001	1259	2.18	0.03
Random	Sardine lab group 1 intercept diff.	0.01				
	Sardine lab group 2 intercept diff.	0.07				
	Sardine lab group 3 intercept diff.	-0.08				
	Squid lab group 1 intercept diff.	0.18				
	Squid lab group 2 intercept diff.	-0.07				
	Squid lab group 3 intercept diff.	-0.11				

F) Species comparison of response latency (τ, s), related to Figure 4A

Effect	Term	Estimate	SE	DF	t-value	p-value
Fixed	Intercept (sardine)	0.34	0.09	38	3.75	< 0.001
	Squid intercept diff.	1.13	0.13	2	8.76	0.01
Random	Sardine lab group 2 intercept diff.	< 0.001				
	Sardine lab group 3 intercept diff.	< 0.001				
	Squid lab group 2 intercept diff.	< 0.001				
	Squid lab group 3 intercept diff.	< 0.001				

G) Species comparison of response accuracy, related to Figure 4B

Effect	Term	Estimate	SE	DF	t-value	p-value
Fixed	Intercept (sardine)	0.15	0.15	38	0.96	0.34
	Squid intercept diff.	-0.03	0.22	2	-0.13	0.91
Random	Sardine lab group 2 intercept diff.	< 0.001				
	Sardine lab group 3 intercept diff.	< 0.001				
	Squid lab group 2 intercept diff.	< 0.001				
	Squid lab group 3 intercept diff.	< 0.001				

Table S1 Results of linear mixed-effects analyses.

Intercept test statistics are on the estimate's difference from 0. Non-intercept estimates for fixed and random effects are differences (diff.) from the intercept. Test statistics for non-intercept fixed effects are on the estimate's difference from the intercept. SE = standard error, DF = degrees of freedom.

Video S1 (separate file)

Example *in situ* footage of a Pacific sardine group (Group 3 in Table 1). We analyzed only the central 2 s of footage (see Materials and methods, Collective organization).

Video S2 (separate file)

Example *in situ* footage of a California market squid group (Group 1 in Table 1). We analyzed only the central 2 s of footage (see Materials and methods, Collective organization).

Video S3 (separate file)

Example lab footage of a Pacific sardine group (Group 1 in Table 2). We analyzed only footage collected immediately before each strobe flash (see Materials and methods, Collective organization).

Video S4 (separate file)

Example lab footage of a California market squid group (Group 1 in Table 2). We analyzed only footage collected immediately before each strobe flash (see Materials and methods, Collective organization).
